# Acceptability and efficacy of the culturally adapted problem management plus intervention for people with disability in Pakistan: a pilot cluster randomized controlled trial

**DOI:** 10.3389/fpsyt.2024.1413809

**Published:** 2025-01-30

**Authors:** Basharat Hussain, Muhammad Tahir Khalily, Ahmed Waqas, Atif Rahman, Ioannis Angelakis, Anum Nisar, Sabir Zaman, Tanveer Akhtar

**Affiliations:** ^1^ Department of Psychology, International Islamic University, Islamabad, Pakistan; ^2^ Department of Psychology and Human Development, Karakoram International University Gilgit, Gligit, Gilgit-Baltistan, Pakistan; ^3^ Faculty of Social Sciences and Humanities, Shifa Tameer-e-Millat University, Islamabad, Pakistan; ^4^ Department of Neurosciences, School of Medical Sciences, Academic Fellow, Universiti Sains Malaysia, Penang, Malaysia; ^5^ Department of Primary Care and Mental Health, Institute of Population Health, University of Liverpool, Liverpool, United Kingdom

**Keywords:** disability and mental health, low-and middle-income countries (LMICs), problem management plus (PM+), community-based interventions, randomized controlled trial, psychological well-being, cultural adaptation of interventions

## Abstract

**Background:**

In Pakistan, the mental health care for persons living with disabilities is insufficient, lacking a holistic approach to address their needs. This research evaluates the adapted PM+’s (IA-PM+) initial efficacy and acceptability in improving mental health among persons living with disabilities, contributing to the integration of mental health services within primary care settings in Pakistan.

**Methods:**

This study employed a two-arm, single-blind cluster randomized controlled trial in a community setting within Union Council Kuri Dolal, Tehsil Gujjar Khan, District Rawalpindi-Pakistan. It compared an Active Treatment group, receiving five sessions of the IA-PM+ intervention, against a Delayed Treatment Control group. A total of 148 participants were randomized. Randomization occurred at the village level, with clusters assigned to either the intervention or control group. The intervention’s effectiveness and participant satisfaction were assessed using a variety of measures, including the GHQ-12, WHODAS 2.0, DASS-21, and the Client Satisfaction Questionnaire, at baseline, the 8th week, and during a 3-month follow-up. Qualitative feedback was gathered at the end of the study to assess the intervention’s acceptability among the participants.

**Results:**

The study achieved a 100% retention rate. Baseline demographics showed a majority male participation with a variety of disabilities predominantly lower limb. Significant improvements were noted in the intervention group across WHODAS scores (Cohen’s d= 0.66), PTSD symptoms (d= 0.75), and DASS scores for depression (d= 0.46), anxiety (d=0.65), and stress (d= 0.47). Similar trends were noted during the follow-up phases. However, life satisfaction scores initially higher in the control group evened out by follow-up, and perceived social support was consistently lower in the intervention group. High levels of client satisfaction were reported in the intervention group, with most participants finding the sessions beneficial and expressing willingness to recommend the service to others. The qualitative interviews revealed that the integration of religious practices, such as associating stress management techniques with Adhan, significantly enhanced the acceptability of the culturally adapted PM+ intervention. Participants reported that this cultural alignment made the coping strategies more relatable and easier to adopt, contributing to improvements in both their mental health and somatic symptoms.

**Clinical Trial Registration:**

https://clinicaltrials.gov/ct2/show/NCT04981522, identifier NCT04981522.

## Introduction

Persons living with disabilities (PLWDs) in Pakistan face significant barriers, not only in terms of physical health but also in accessing adequate mental health support. Globally, an estimated one billion people, or 16% of the world population, experience some form of disability, with approximately 200 million individuals facing significant functional difficulties (1, 2). The prevalence of disabilities is notably higher in low- and middle-income countries (LMICs) due to factors such as aging populations, increased rates of chronic health conditions, and limited healthcare resources (2, 3). The mental health of PLWDs is an area of significant concern, with evidence suggesting that they are at a higher risk of developing psychological issues compared to their non-disabled counterparts. This vulnerability is attributed to several factors including social isolation, stigma, discrimination, and the physical and emotional stresses associated with disability (4, 5). Despite this heightened risk, the healthcare response in Pakistan remains inadequate, lacking a comprehensive strategy that integrates interdisciplinary approaches to address the psychiatric ailments prevalent among this population (6, 7).

Recognizing the critical need for targeted mental health interventions, this study aimed to adapt and implement the World Health Organization’s Problem Management Plus (PM+) intervention within the Pakistani context. PM+ intervention is a low-intensity psychological intervention designed to address common mental health issues such as depression, anxiety, and stress, particularly in settings with limited mental health resources. The intervention employs paraprofessionals to deliver support, making it a viable option for community-based mental health care in LMICs (3, 8). By leveraging a community-based approach and integrating cultural and religious dimensions, the adapted PM+ programme, hereafter referred to as the indigenously adapted Problem Management Plus (IA-PM+) intervention, represents a pioneering effort to provide holistic and accessible mental health support to one of the most vulnerable segments of the population. Moreover, this study contributes to the broader discourse on the integration of mental health care within primary health settings, advocating for a model that respects the biopsychosocial dimensions of health and well-being. This study aimed to evaluate the effectiveness of the indigenously adapted Problem Management Plus (IA-PM+) in reducing psychological distress among persons living with disabilities, measured by improvements in mental health outcomes such as anxiety, depression, and stress, through validated psychological scales. Furthermore, using qualitative methods, it also aims to explore the acceptability of the IA-PM+ intervention, focusing on participant engagement, satisfaction, cultural relevance, and the feasibility of delivering the intervention by community-based inclusive development workers, as assessed through qualitative interviews and feedback from participants and facilitators.

## Methods

### Research setting

The current study consists of a two-arm, single-blind cluster randomized controlled trial (c-RCT) conducted in a community setting in Union Council Kuri Dolal, Tehsil Gujjar Khan, District Rawalpindi. The clinical trial was registered prior to the recruitment commencing with US National Institute of Health (US National Library of Medicine) on 29 July 2021, under registration number NCT04981522 (https://clinicaltrials.gov/ct2/show/NCT04981522).

### Participants

This community-based intervention was conducted in two union councils of Gujjar Khan encompassing 08 villages (54 hamlets), comprising a homogenous population. To recruit participants in the trial, screening was conducted using the General Health Questionnaire (GHQ-12) and WHO Disability Assessment Scale (WHODAS 2.0) and screening checklist. Those scoring positive for psychological distress on the GHQ-12 (cut-off ≥ 3) and WHODAS 2.0 (cut-off 25), then underwent further screening.

The study recruited individuals who met specific inclusion criteria: they scored above 16 on the PSYCHLOPS questionnaire (Psychological Outcome Profiles Questionnaire), were over 18 years old, could effectively engage with the research questions, were willing to provide informed consent, and had been living with permanent physical disabilities (e.g. hemiplegia, paraplegia, blindness, deafness, stroke leading to locomotor disabilities) for more than six months. The study excluded individuals who were temporary residents or lived outside the designated study area. Moreover, we excluded those with pre-existing diagnoses of conditions such as personality disorders, substance abuse, suicidal ideation, epilepsy, dementia, autism, Down syndrome, cerebral palsy, learning disabilities, intellectual disabilities, or communication disabilities, or were unable to engage with or respond to the research questions effectively. These individuals were excluded because PM+ was originally designed for the management of common mental disorders and is not recommended for severe mental disorders, neurological conditions, or personality disorders.

The PSYCHLOPS was used to establish baseline severity levels of distress (9), encompassing three key domains: problems (assessed through two questions), functioning (assessed through one question), and well-being (assessed through one question). This questionnaire is scored on a six-point scale from 0 to 5, where higher scores indicate more severe issues. It The PSYCHLOPS has been found to exhibit strong internal consistency, robust convergent validity when compared with other psychological distress measures, and a high level of sensitivity to capturing changes over time (9).

### Randomization

Randomization was executed by an independent statistician, uninvolved in other study activities, using the Random Allocation software (10). Village clusters were randomized in a 1:1 ratio employing a permuted-block method to either the IA-PM+ intervention or the Delay Treatment Group. This was conducted at the village level, aligning with the community-based nature of the available rehabilitation services and the geographic setting, thereby justifying the use of a Cluster Randomized Control Trial (c-RCT) design.

### Intervention details

The c-RCT comprised two intervention groups: Active Treatment (AT; with 05 sessions of IA-PM+ intervention) and Delayed Treatment Control (DTC; treatment delayed until the last follow-up).

#### Intervention

The World Health Organization’s Problem Management Plus (PM+) intervention, developed to address common mental health problems in settings of adversity (8). It has been validated for use in general population in Pakistan (11). However, its applicability to the context of person with physical disabilities has not been explored or validated. This adaptation was crucial, as cultural congruence plays a significant role in the acceptability and effectiveness of mental health interventions. Adaptations made to the core components of the PM+ are presented in **Table 1**.

**Table 1:** Core components of IA-PM+.

Session	Core component	Detail
Session-01	Stress management	Islamic stress management techniques, like prayer (Salah) and mindfulness (Dhikr), aligning with the cognitive-behavioral principles of PM+ while adding a spiritual dimension (11).
Session-02	Problem solving	Problem-solving within the IA-PM+ is framed through Islamic concepts of patience (Sabr) and reliance on God (Tawakkul), providing a culturally coherent approach to challenges (12).
Session-03	Behavioural activation	Activities promoted by this component are chosen for their cultural and religious significance, fostering a sense of achievement and community integration (7, 8).
Session-04	Social support	This component emphasizes the role of the Muslim community (Ummah) in offering support, leveraging Islamic teachings on communal care and solidarity (7, 8).
Session-05	Resilience	Reflects the Islamic emphasis on continuous self-improvement (Tazkiyah) and resilience, encouraging participants to maintain progress and prepare for future challenges with a spiritual outlook (7, 8).

The indigenously adapted version of PM+, IA-PM+, was adapted to align with the cultural and religious sensibilities of the Pakistani population. This intervention incorporated cognitive coping and behavioural coping strategies grounded in the Islamic principles and the local cultural practices, aiming to enhance its relevance and impact on the target population (13–15). The IA-PM+ acknowledges the Islamic view of disability and mental health as tests of faith and opportunities for growth, advocating for a respectful and inclusive support system that recognizes the potential and dignity of every individual, in line with Islamic teachings on disability (16, 17).

The IA-PM+ intervention comprised five sessions, aimed at training the participants in techniques to manage their emotional problems. Each session lasted 90 minutes. Since persons with disabilities have complex and specific needs, therefore, the IA-PM+ was designed for individual session format rather than group session.

*Delay Treatment Control (DTC):* The participants who randomized into the DTC group received routine rehabilitation services based on the medical and social models from Community Based Inclusive Development Workers (CBWs). The participants in the DTC arm received routine care until the end of the last follow-up and were not offered the IA-PM+ intervention for the duration of the study.

### Training and supervision

The implementation of the Indigenously Adapted Problem Management Plus (IA-PM+) by trained community-based inclusive development workers (CBWs) was a strategic response to the scarcity of mental health professionals in Pakistan. CBWs were chosen to deliver the intervention due to the limited availability of trained psychologists and social workers, particularly in rural areas. These health workers are already integrated into the existing healthcare infrastructure in Pakistan. By employing paraprofessionals to deliver PM+ (a low-intensity psychological intervention), this approach effectively addresses mental health issues effectively within community settings. This strategy provides holistic and accessible mental health support to one of the most vulnerable segments of the population, offering a viable model for community-based mental health care in low- and middle-income countries (LMICs). The training integrated Islamic teachings and local cultural practices, enhancing the relevance and accessibility of mental health support to align with the community-focused approach of PM+.

To provide psychological intervention, ten trained community-based workers (CBWs) within the selected area provided low intensity indigenous psychological intervention to PLWDs. For this study, individuals from the Basic health unit and community based inclusive development centre were recruited for the IA-PM+ training and intervention. These individuals were selected based on their qualifications (equivalent to 12th grade), basic communication skills, interest, motivation and commitment to work.

Health workers underwent a comprehensive 10-day training programme covering the fundamentals of IA-PM+ intervention and basic counselling techniques. Post-training, these workers completed two practice cycles, each involving the examination of two clients over five sessions within a two-week timeframe. This hands-on experience was supported by rigorous supervision to maintain protocol fidelity. The training regimen for IA-PM+ included both classroom instruction and field practice, emphasizing the practical application of Islamic and cultural adaptations in therapeutic contexts. This training was augmented by an apprenticeship model of supervision, where trainees were closely monitored by seasoned mental health professionals. This supervision ensured that the training adhered to the program’s standards while respecting cultural sensitivities.

Supervision was conducted by individuals who had not only completed the IA-PM+ training themselves but had also undergone an additional two days of specialized training in supervision techniques. These supervisors were tasked with ensuring the adherence to the IA-PM+ protocols through routine supervision, weekly group sessions to discuss client progress and challenges, and random session attendance to check on treatment fidelity using a standardized checklist. Supervisors also facilitated self-evaluation among IA-PM+ providers, encouraging reflection and discussion during weekly supervision meetings.

### Outcomes

The baseline assessment was completed to evaluate baseline symptoms and associated features, followed by an 8-week outcome assessment and a 3 months follow-up. All assessments were conducted by trained health professionals who received four days of training in research ethics, interview techniques and assessment battery designed for the clinical trial. The outcome assessors were blind to the allocation status of the study population. Outcomes were measured at baseline (Pre IA-PM+ intervention), post IA-PM+ intervention (8 weeks after baseline) and follow-up (20 ± 1 weeks of the baseline). Urdu translations of psychometric rating scales were used.

General Health Questionnaire (GHQ-12): GHQ-12 is a 12-item self-report Likert scale that assesses the level of psychological distress. Translation procedures for the GHQ-Urdu have been described in previous studies in Pakistan (18, 19). In the current study, the Urdu version of GHQ-12 demonstrated adequate psychometric properties with high internal consistency (Cronbach’s α = 0.92). Additionally, it also yielded a unidimensional factor structure explaining 57% of the variance in the scale, with strong factor loadings ranging from 0.59 for item 3 to 0.86 for item 7. Increasing scores on the GHQ-12 correspond to poorer outcomes.

#### WHO Disability Assessment Scale (WHODAS 2.0)

WHODAS 2.0 is a self-report scale used to assess the functionality of individuals experiencing difficulties associated with their illness across six domains of functioning, including mobility, self-care, cognition, getting along, participation and life activities. It employs five-point Likert scale to record experiences over the last 30 days. The Urdu version of 12-item WHODAS 2.0 was used in this study, demonstrating acceptable psychometric properties with high internal consistency (Cronbach’s α= 0.84). In addition to screening, it was also used as a secondary outcome (20). Field testing was conducted by the World Health Organization in 19 countries, yielded a stable factor structure, and excellent test-retest reliability (intraclass correlation coefficient: 0.98). It demonstrated strong concurrent validity for patient classification when compared to established disability measurement tools, aligns well with Rasch scaling properties across diverse populations, shows high responsiveness, indicating sensitivity to change (21). Increasing scores correspond to poorer outcomes.

#### Depression anxiety and stress scale (DASS-21)

The DASS-21 is a 21-item subjectively rated scale measured on a four-point Likert scale. It is widely used to assess the symptoms of depression, anxiety, and stress among the study population (22). It has shown excellent psychometric properties in a recent study of 2,000 Pakistanis, yielding high internal consistency for each of the subscales, a three-dimensional factor structure demonstrating acceptable goodness of fit indices, and responsiveness to change (23). In this study, the DASS-21 demonstrated good psychometric properties with high internal consistency (Cronbach’s α = 0.95) (24). Increasing scores correspond to poorer outcomes.

#### Multidimensional Scale of Perceived Social Support (MSPSS)

The MSPSS measures perceived social support and consists of 12 items, with responses ranging from very strongly disagree (coded as 1) to very strongly agree (coded as 7). These items encompass support from family, friends, and other significant relationships. The total score for all items ranges from 12 to 84, with higher scores indicating higher perceived social support. It demonstrated high internal consistency (Cronbach’s α = 0.92) in this study. Akhtar et al., has previously reported good cross-cultural properties, internal consistency and factor validity of MSPSS in a sample of Pakistani women (25, 26). Increasing scores correspond to favourable outcomes.

#### Satisfaction with Life Scale (SWLs)

SWLs is a 5-items Likert scale designed to measure cognitive judgments of one’s satisfaction with life on a seven-point scale, with responses ranging from ‘strong disagreement = 1’ to ‘strong agreement = 7’. The Urdu version of SWLs demonstrated adequate psychometric properties with high internal consistency (Cronbach’s α = 0.84) (27). Barki et al., in a recent study of 120 Pakistanis, showed that the SWL yielded a one-factor model demonstrating excellent fit indices in Confirmatory Factor Analysis. Increasing scores correspond to higher satisfaction with life.

#### Posttraumatic stress disorder (PTSD) symptoms Checklist (PCL-C)

PCL-C is a 17-item scale used to measure the symptoms associated with traumatic events experienced during the past week. Item responses range from 1 to 5, with the total score for all items ranging from 17 to 85. It demonstrated a high internal consistency (Cronbach’s α = 0.95) in this study. The scale also exhibited a unidimensional factor structure using factor analysis with principal axis factoring. It explained a cumulative variance of 56.60%, with strong factor loadings ranging from 0.59 (Item 1) to 0.83 (Item 17) (12). Increasing scores correspond to poorer outcomes.

#### Client Satisfaction Questionaire (CSQ-8)

CSQ is a 8-item brief scale used to measure client satisfaction with the services. In this study, the Urdu version of CSQ was used to assess the satisfaction of PLWDs, demonstrating adequate psychometric properties with high internal consistency (Cronbach’s α = 0.89). This questionnaire was administered upon the completion of intervention sessions (28). Increasing scores correspond to favourable outcomes.

#### Acceptability of the intervention

Qualitative interviews were conducted at the conclusion of the study to evaluate the acceptability of the intervention among the participants.

### Ethical considerations

Ethical approval was obtained from the Bioethics Committee of the International Islamic University Islamabad (September 10, 2021). Furthermore, informed consent was also sought from the participants by ensuring their privacy and confidentiality on the matters. Additionally, the researcher secured permission from the authors to utilize the scale in the current study.

### Quantitative data analysis

Sample size calculation was based upon the PM+ based intervention for psychological problems (29). To determine the size of the study effect, a sample of 128 participants was required to achieve 80% of statistical power. However, considering the expected dropout rate of about 15%, the study aimed to include 148 participants, with an equal proportion of randomization to both groups (IA-PM+ = 74: DTC = 74).

All statistical analyses were conducted using the Statistical Package for Social Sciences Version 29 (SPSS 29.0). Means and standard deviations (SD) were presented for quantitative variables, while frequencies and percentages (%) were reported for categorical variables. Our study was structured around a two-group design, including an intervention group and a delay treatment group. To investigate the effectiveness of the intervention, independent sample t-tests were performed to evaluate between-group differences (TAU – IA-PM+) across primary and secondary outcomes measured post-intervention and follow-up. Cohen’s d were further calculated to present effect size of the intervention. To ascertain the within group differences, a series of paired sample-test was run for all primary and secondary outcomes.

To assess the intervention’s effectiveness, linear mixed effects models (LMM) were employed using the MIXED procedure in SPSS 29.0. This approach accounted for clustering at the village level and adjusted for baseline covariates, including age, gender, education, and baseline outcome scores, ensuring robust estimation of the intervention’s effects. Multicollinearity was assessed using variance inflation factors (VIF), confirming no significant issues. Residuals were checked for normality, and the models were found robust to potential deviations, given the sample size and clustering adjustments.

### Qualitative data collection and analysis

The qualitative component of this study involved in-depth interviews with 10 participants who received the IA-PM+ intervention. These interviews were conducted at the end of the study to assess the intervention’s acceptability. Participants were selected based on their willingness to share their experiences and provide detailed feedback. The interviews aimed to capture a broad range of perspectives on the intervention’s impact, the relevance of the content, and the participants’ overall satisfaction.

The qualitative data were analysed using a combination of thematic content analysis (30) and interpretative phenomenological analysis (IPA) (31). This dual approach provided a comprehensive exploration of the participants’ experiences and perceptions. Thematic content analysis involved transcribing the interviews verbatim and systematically coding them (30). The coding process focused on identifying significant statements, phrases, and recurring themes across the interviews. These themes were then categorized into broader domains to summarize the collective experiences of the participants, allowing the researchers to identify common patterns and insights regarding the intervention’s acceptability (30).

In addition, IPA was used to delve deeper into the individual experiences of the participants. This approach focused on understanding how participants made sense of their experiences with the intervention (31). The analysis involved a detailed examination of each participant’s narrative to capture the essence of their personal experiences. Through IPA, the researchers explored the meanings and significance that participants attributed to the intervention, providing a nuanced understanding of its impact on their mental health and well-being (31).

## Results

### Participant recruitment

**Figure 1:**
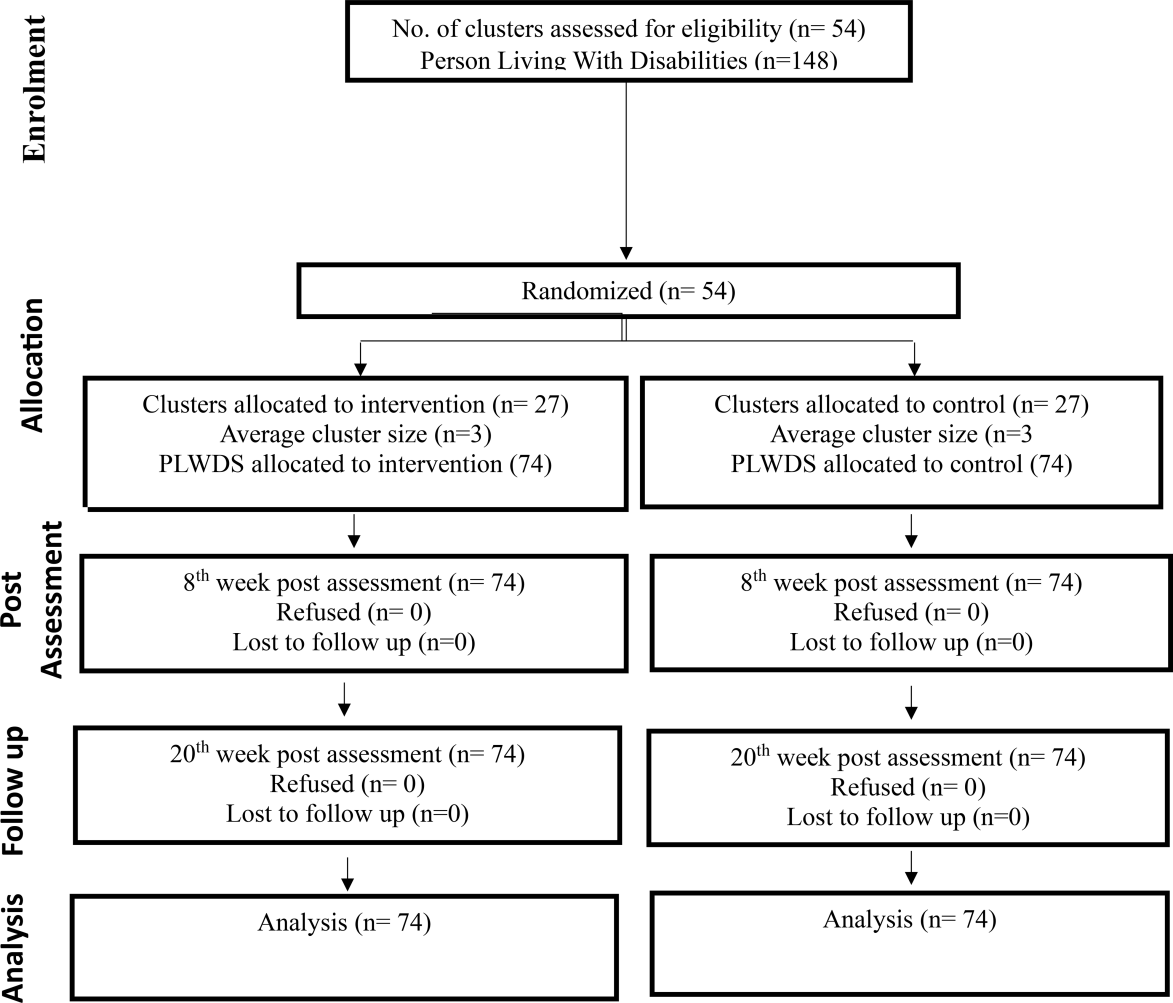
Consort flow diagram of the trial.

**Figure 1** presents the CONSORT flow diagram for participant recruitment. A total of 2,169 individuals in 8 villages were approached, out of which 227 participants met the inclusion criteria. 148 agreed to participate in the trial and were randomly allocated either to the intervention or control/TAU based on their villages. Subsequently, 74 participants (out of 4 villages) were randomly allocated to intervention and 74 participants (out of 4 villages) were allocated to control/TAU. All the participants who were randomised, completed the study (n = 148) indicating an excellent retention rate (100%). All participants in the intervention group attended the 5 sessions of IA-PM+ intervention.

### Demographic characteristics

**Table 1** presents the baseline demographic characteristics of the study variables. The study participants were equally divided into two groups: the control group consisting of 74 (50.0%) participants and the Intervention group also consisting of 74 (50.0%) participants. Furthermore, majority of the participants were male accounting for 81 (54.7%) individuals, compared to 67 female (45.3%). The mean age of the participants was 37.55 (11.40) years and mean duration of disability was 18.65 (14.79) years (**Table 2**).

**Table 2:** Baseline socio-demographic characteristics of the study population (n=148).

Category	Subcategory	Control Group (n=74)	Intervention Group (IA-PM+, n= 74)	Total
Socio-demographic Characteristics				
Gender	Male	-	-	81 (54.7%)
	Female	-	-	67 (45.3%)
Family System	Nuclear	-	-	78 (52.7%)
	Joint	-	-	70 (47.3%)
Education	Illiterate	-	-	57 (38.5%)
	Primary	-	-	58 (39.2%)
	Middle	-	-	26 (17.6%)
	Matric	-	-	3 (2.0%)
	Intermediate	-	-	4 (2.7%)
Marital Status	Unmarried	-	-	56 (37.8%)
	Married	-	-	92 (62.2%)
	Age	38.95 ± 10.65	36.15 ± 12.02	37.55 ± 11.40
	Duration of Disability	-	-	18.65 ± 14.79
Disability Characteristics				
Disability Type	Lower Limb (Mobility)	-	-	84 (56.8%)
	Vision	-	-	13 (8.8%)
	Hearing	-	-	2 (1.4%)
	Upper Limb (Independent Living)	-	-	7 (4.7%)
	Locomotor (Self-care)	-	-	42 (28.4%)
Disability Aetiology	Congenital	-	-	60 (40.5%)
	Accident	-	-	30 (20.3%)
	Infection	-	-	33 (22.3%)
	Senile	-	-	13 (8.8%)
	Stroke	-	-	12 (8.1%)
Clinical Baseline Data				
	GHQ	20.91 ± 6.41	21.83 ± 6.71	-
	WHODAS	35.99 ± 8.21	36.36 ± 7.72	-
	DASS - Depression	11.54 ± 4.11	12.45 ± 4.05	-
	DASS - Anxiety	11.74 ± 3.75	12.21 ± 3.19	-
	DASS - Stress	11.93 ± 3.49	13.07 ± 3.96	-
	PCL-C	20.81 ± 11.61	21.74 ± 13.21	-
	MSPSS	46.24 ± 13.48	46.84 ± 13.56	-
	SWLs	18.55 ± 4.78	16.50 ± 4.83	-

We observed a variety of disability types among participants, with a predominant prevalence of lower limb (mobility) disabilities, affecting 84 individuals (56.8%). Other disabilities included upper limb (independent living) disabilities in 7 participants (4.7%), vision disabilities in 13 individuals (8.8%), hearing disabilities in 2 participants (1.4%), and locomotor (self-care) disabilities in 42 individuals (28.4%). The causes of these disabilities varied, with congenital factors leading in 60 cases (40.5%), followed by accidents in 30 individuals (20.3%), infections in 33 participants (22.3%), age-related issues in 13 individuals (8.8%), and strokes in 12 participants (8.1%). Notably, none of the participants reported a history of suicidal ideation or severe mental illness.

### Outcomes

For WHODAS scores, the IA-PM+ intervention demonstrated significant improvements both post-intervention and at follow-up, with adjusted mean differences indicating a reduction in disability severity (post-intervention: [5.92, 95% CI: 3.01 to 8.83, d = 0.66, p <.001]; Follow-up: [7.80, 95% CI: 4.62 to 10.98, d = 0.80, p <.001]). PTSD symptoms, measured by PCL-C, indicated significant improvements post-intervention with further enhancements at follow-up (post-intervention: [8.76, 95% CI: 4.94 to 12.57, d = 0.75, p = .03]; Follow-up: [7.51, 95% CI: 3.57 to 11.46, d = 0.62, p <.001]) (**Table 3**).

**Table 3:** Intervention effect sizes for primary and secondary outcomes at 6^th^ Week and at 20^th^ Week follow-up (n=148).

Variable	6th Week Outcome Adjusted Mean Difference (95% CI) TAU – IA-PM+	Effect Size (Cohen’s d)	Statistics t, p	20th Week follow-up Adjusted Mean Difference (95% CI) TAU – IA-PM+	Effect Size (Cohen’s d)	Statistics t, p
WHODAS	5.92 (3.01, 8.83)	0.66	4.02,.001	7.80 (4.62, 10.98)	0.80	4.86, .001
DASS	5.39 (2.20, 8.58)	0.55	3.34,.001	5.39 (2.20, 8,58)	0.55	3.34, .001
Depression	1.61 (0.47, 2.75)	0.46	2.79,.006	3.22 (2.07, 4.36)	0.91	5.55, .001
Anxiety	2.09 (1.04, 3.15)	0.65	3.93,.001	2.65 (1.75, 3.55)	0.96	5.81, .001
Stress	1.69 (0.51, 2.87)	0.47	2.83,.005	2.20 (1.08, 3.32)	0.64	3.91, .001
PCL-C	8.76 (4.94, 12.57)	0.75	4,54,.03	7.51 (3.57, 11.46)	0.62	2.16, .001
MSPSS	-4.22 (-7.97, -0.46)	0.37	2.22,.01	-4.34 (-8.31, -.038)	0.35	1.72, .03
SWLs	2.05 (0.49, 3.62)	0.43	2.60,.001	-1.12 (-2.41, -0.17)	0.28	3.77, .09

IA-PM+, Indigenously adapted Problem Management Plus; WHODAS, WHO Disability Assessment Schedule; DASS, Depression, Anxiety and Stress Scale; PSP, Perceived Social Support; PCL-C, PTSD Checklist Civilian version; MSPSS, Multidimensional Scale of Perceived Social Support; SWLs, Satisfaction with Life Scale.

DASS scores showed significant improvements at both time points, underlining the intervention’s efficacy in reducing symptoms of depression, anxiety, and stress (post-intervention and Follow-up: [5.39, 95% CI: 2.20 to 8.58, d = 0.55, p <.001]). Depression scores, as part of the DASS subscales, decreased significantly post-intervention and showed further improvement at follow-up (post-intervention: [1.61, 95% CI: 0.47 to 2.75, d = 0.46, p = .006]; Follow-up: [3.22, 95% CI: 2.07 to 4.36, d = 0.91, p <.001]). Anxiety outcomes experienced significant reductions post-intervention with further improvements at follow-up (post-intervention: [2.09, 95% CI: 1.04 to 3.15, d = 0.65, p <.001]; Follow-up: [2.65, 95% CI: 1.75 to 3.55, d = 0.96, p <.001]). Stress outcomes also showed significant improvements post-intervention, sustained at follow-up (post-intervention: [1.69, 95% CI: 0.51 to 2.87, d = 0.47, p = .005]; Follow-up: [2.20, 95% CI: 1.08 to 3.32, d = 0.64, p <.001]).

The control group reported better satisfaction with life scores immediately post-intervention ([2.05, 95% CI: 0.49 to 3.62, d = 0.43, p = .001]), but this advantage dissipated by the follow-up period ([-1.12, 95% CI: -2.41 to -0.17, d = 0.28, p = .09]), indicating no long-term difference between the groups in terms of life satisfaction. Perceived social support appeared to be lower in the intervention group, as indicated by the negative effect sizes at both post-intervention and follow-up (post-intervention: [-4.22, 95% CI: -7.97 to -0.46, d = -0.37, p = .01]; Follow-up: [-4.34, 95% CI: -8.31 to -0.38, d = -0.35, p = .03]).

These results were maintained in linear mixed model analyses. Results for linear mixed models are presented in **Supplementary Tables 1**–**9**. Detailed descriptive statistics for outcome assessments are provided in **Supplementary Table 10**.

Similar pre-post trends were noted with statistically significant improvements noted in the intervention group at baseline (**Supplementary Table 11**). Effect sizes (Cohen’s d) ranged from 0.54 for PCL-C to 1.35 for anxiety subscale of the DASS. Negative effect sizes were noted for perceived social support (Cohen’s d= -0.27).

These effect sizes were maintained at follow up (**Supplementary Table 11**) with Cohen’s d ranging from 0.66 for PCL-C to 1.76 for depression subscale of the DASS. Scores worsened for perceived social support (d= -0.39) and life satisfaction (d= -0.71).

### Acceptability of the intervention

#### Client satisfaction questionnaire

The analysis revealed that the intervention group reported significantly higher levels of client satisfaction compared to the control group, with a mean difference of -0.77 (95% CI: -1.18 to -0.36, p <.001), and a large effect size indicated by Cohen’s d of 0.61. Responses CSQ-8 showed that the majority of respondents rated the quality as good (68.2%) with a significant portion finding it fair (31.8%), while none considered it poor or excellent. Most participants felt they received the type of service they wanted, with 82.4% affirmatively responding “Yes, generally,” and a smaller group (14.9%) feeling they definitely got what they wanted. Regarding the service meeting their needs, more than half (56.1%) felt that only a few of their needs were met, yet a notable percentage (33.1%) believed most of their needs were addressed. A substantial majority would recommend the service to a friend in need (75.7% “Yes, I think so” and 21.6% “Yes, definitely”), indicating a positive overall perception. Satisfaction with the amount of help received was high, with 87.2% mostly satisfied and 10.1% very satisfied. When asked if the services helped deal with their problems, 48.6% acknowledged some help, and 21.6% felt a great deal of help was provided, although 29.7% didn’t notice significant assistance (**Supplementary Table 12**).

### Insights from qualitative interviews

#### Development of cognitive coping skills

The qualitative interviews regarding the intervention revealed its high acceptability among participants, primarily attributed to the development of cognitive and behavioural coping skills. A service user opined that, ‘We have learned that we should not conceal our worries and tension. It’s important to share them with a trustworthy person; this will help us relax. I acquired this knowledge from the Salamti program” (Participant’s response)’.

Another participant shared how the intervention helped with their somatic symptoms, ‘“I had many health issues, including eating problem, sleep disturbances and feeling weak, but now I have forgotten all these things” (Participant’s response).

#### Improved help-seeking

Participants expressed valuable insights, noting the importance of not concealing worries and the benefit of sharing concerns with trustworthy individuals, as one stated, “It’s important to share them with a trustworthy person; this will help us relax.” Another service user shared about modifications in their help seeking behaviour, ‘I have learned that we should seek help from experienced individuals without feeling shy. if we openly discuss our genuine problems, people will be willing to assist us’ (Participant’s response).

#### Cross-cultural adaptation

The intervention, referred to as the “Salamti program,” was praised for teaching participants to seek help without hesitation and for introducing effective stress management techniques, including those associated with religious practices like Adhan. Many reported significant improvements in health issues such as eating problems, sleep disturbances, and a general sense of weakness, highlighting the practical applications of the program like the use of a calendar to encourage social and religious engagement. A health worker shared that, ‘Health workers shared how associating coping skills with religious practices was useful, ‘The patients were happy to learn stress management techniques and they associated the breathing exercises with Adhan” (Response of a health worker)’.

#### Barriers to participation

Initially, there was hesitancy to participate due to negative perceptions of the program.

However, this skepticism waned as participants recognized the program’s benefits for their well-being; a service user noted that “At first, I was hesitant because people had said negative things about this program, but now I realize that this program is beneficial for our health” (Participant’s response).

This sentiment was echoed by the health workers who noted a shift in participants’ openness over time “At the beginning of the program, people were not very open, but later on, they started opening up when they realized that this program is beneficial for their wellbeing” (Health worker).

#### Skill development of health workers

The training component of the intervention was also well-received, particularly for its role in skill development among delivery agents. Health workers and supervisors reported gaining insights into somatic symptoms and observed significant health improvements in patients following the intervention, underscoring the program’s comprehensive approach to mental health and well-being: “In training, we learned about a type of illness called somatic symptoms. We observed that many patients reported somatic symptoms and with this intervention, we observed significant changes in their health” (Response of the supervisor)’.

## Discussion

### Summary of findings

This study evaluated an indigenously adapted psychological intervention based on the World Health Organization’s Problem Management Plus (IA-PM+) to mitigate psychological distress (anxiety, depression, stress and trauma) faced by individuals living with physical disabilities in Pakistan. The intervention utilized cognitive and behavioural strategies aligned with religious and spiritual principles, to offer a culturally resonant approach to mental health support. The intervention was delivered through task-sharing by healthcare workers, leveraging integration within the primary healthcare network and community-based non-governmental organizations. The intervention led to improvements in symptoms of anxiety, depression, and stress and trauma. Qualitative feedback from participants and healthcare workers further reinforced the intervention’s acceptability and effectiveness. The emphasis on cognitive and behavioural coping skills resonated with participants, who reported a post-intervention sense of empowerment and well-being.

### Acceptability of the intervention

Pakistan’s health system presents with several challenges for PLWDs, marked by a substantial physical and mental health treatment gap and prevailing cultural stigmas (7, 32). This study aligns with global endeavours to bridge the mental health treatment gap, particularly for vulnerable populations like PLWDs, who require a more inclusive and comprehensive healthcare.

The IA-PM+ intervention emerges as a simple, adaptable, and short-duration intervention deliverable by non-specialists, which can be integrated within the primary healthcare and NGO sectors. The IA-PM+ intervention offers a scalable and effective strategy as it leverages task-sharing and culturally sensitive approaches, which align with global recommendations to address mental health disparities (14).This intervention complements the growing list of psychosocial interventions in Pakistan. These include the WHO-endorsed Thinking Healthy Programme for perinatal depression (33), Learning through Play Plus for perinatal depression and child development (34), PM+ for adults with mood and anxiety disorders in post-conflict areas of Pakistan (14) and the Child School Mental Health Programme, delivered by teachers to support child and adolescent mental health (35). Each has been adapted and integrated, using task-sharing to meet the country’s mental health needs effectively.

### Effectiveness of the intervention

Overall, the IA-PM+ intervention proved highly effective in addressing both functional and psychological outcomes, with lasting improvements observed, suggesting its potential as a sustainable intervention for this population. Our results are comparable to the findings of previously published literature on scalable psychosocial interventions. For instance, a recent meta-analysis by Schäfer et al. (36) demonstrated small to moderate favourable effects of the WHO’s Problem Management Plus (PM+) and Step-by-Step (SbS) interventions on distress indicators, such as anxiety (SMD = –0.51), depression (SMD = –0.46), PTSD (SMD = –0.34), and functional impairment (SMD = –0.36). In line with these findings, our study showed significant improvements across similar domains, with medium to large effect sizes, particularly in reducing disability severity (post-intervention: d = 0.66, follow-up: d = 0.80), PTSD symptoms (post-intervention: d = 0.75, follow-up: d = 0.62), and emotional distress, including depression (follow-up: d = 0.91) and anxiety (follow-up: d = 0.96). The larger effect sizes observed in our study could be attributed to the cultural adaptation of the intervention, enhancing its relevance and impact for individuals with physical disabilities in our population.

The intriguing findings regarding the decrease in perceived levels of social support and life satisfaction among the intervention group warrant a nuanced exploration. One plausible explanation for this phenomenon lies in the nature of the psychological intervention itself, particularly its focus on developing coping skills and fostering self-awareness among participants (37). The intervention, designed to empower individuals with disabilities by enhancing their coping mechanisms and psychological resilience, may have inadvertently heightened their awareness of the discrepancies between their current social support systems and the ideal level of support necessary for optimal well-being (38). Consequently, this could manifest as a perceived decrease in social support, not necessarily because actual support diminished.

Similarly, the decrease in life satisfaction could be attributed to the introspective processes that are encouraged by the intervention. As participants engage in self-reflection and gain insights into their mental and emotional landscapes, they may reassess their life circumstances through a more critical lens (39). This introspection, while valuable for personal growth and the development of coping strategies, might temporarily affect their overall satisfaction with life. The process of acknowledging and confronting one’s psychological challenges can be inherently unsettling, leading to a transient phase where life satisfaction appears to wane as individuals navigate the complexities of their internal experiences and external realities (27).

It is also worth considering the role of perceived measures in capturing these shifts. The subjective nature of perceived social support and life satisfaction measures means they are inherently influenced by participants’ interpretations of their experiences (25, 27). As such, these measures are sensitive to changes in participants’ internal states and perceptions, which are likely to be impacted by the intervention’s emphasis on self-awareness and coping skills development.

### Cultural adaptation: findings from qualitative analyses

The cultural adaptation of psychosocial interventions, such as the Problem Management Plus (PM+) program in this study, is essential to enhance their relevance and effectiveness, particularly in non-Western settings. The literature highlights how cultural differences—such as communal versus individualistic values and spiritual versus materialistic beliefs—can shape the reception of interventions (40–44). For instance, Scorzelli (1994) emphasized that many traditional psychotherapy models, developed by white Western males, often conflict with the cultural values of clients from non-Western backgrounds, necessitating tailored approaches (45). In the current study, the PM+ intervention’s integration of religious practices, such as linking stress management techniques with Adhan, significantly enhanced its acceptability and relatability for participants. This finding aligns with prior research indicating that non-Western populations may find interventions more effective when they resonate with spiritual and community-oriented values (46, 47). Furthermore, the role of religious and cultural practices in fostering coping skills was well-supported by service users and health workers alike, suggesting that the program’s cultural sensitivity was key to its success. This supports the broader argument that culturally adapted psychosocial interventions—by aligning with participants’ worldviews, such as religious fatalism and community interdependence—are more likely to achieve engagement and positive outcomes (47, 48). Therefore, incorporating cultural elements into interventions not only makes them more relatable but also enhances their efficacy, as evidenced by the positive reception and health improvements observed in this trial.

### Suggestions and recommendations

The impact of the link between physical disability and mental health is not limited to the individuals with disabilities themselves but also extends to their caregivers and family members. Caregivers of individuals with physical disabilities may face increased stress, burden, and mental health challenges due to the demands of caregiving, financial strain, and social isolation. The current study focused solely on PLWDs. Therefore, future studies should consider designing an intervention plan to support the mental health needs of family members of PLWDs as well.

In light of the observed changes in perceived social support and life satisfaction among participants, the role of caregivers emerges as a pivotal factor in the therapeutic landscape. The nuanced dynamics uncovered in the intervention group underscore the necessity of integrating caregivers into the therapeutic process. By involving caregivers in the intervention, they can gain a deeper understanding of the psychological challenges faced by those they care for, and be equipped with effective strategies to enhance their supportive capabilities. Future research could further elucidate these dynamics by exploring the long-term trajectories of perceived social support and life satisfaction post-intervention, examining whether these initial decreases give way to increased well-being as individuals consolidate their coping skills and adapt to their enhanced awareness.

This study demonstrates the feasibility of the IA-PM+ intervention. To further assess its practicality, a well-powered hybrid trial focusing on implementation, alongside a cost-effectiveness analysis, is recommended. Such research should explore implementation barriers, real-world effectiveness, and cost efficiency to inform potential broader adoption.

## Conclusion

This pilot trial demonstrated that the PM+ intervention adapted for people living with disabilities in Pakistan, is efficacious in alleviating common mental health problems such as depression, anxiety, and post-traumatic stress symptoms. Future appropriately powered trials are recommended for definitive evidence.

## Data Availability

The raw data supporting the conclusions of this article will be made available by the authors, without undue reservation.
