# Supplementary material for: Acceptability and efficacy of the culturally adapted problem management plus intervention for people with disability in Pakistan: a pilot cluster randomized controlled trial
Source: Front Psychiatry. 2025 Jan 30;15:1413809. doi: 10.3389/fpsyt.2024.1413809 (PMC11841386; doi:10.3389/fpsyt.2024.1413809)
Supplement: Supplementary file 1 [file Table1.docx]

**Summary results for mixed effects model**

The results from the Linear Mixed Effects models demonstrate the effectiveness of the IA-PM+ intervention across various outcomes. For disability scores post-intervention and at long-term follow-up, recipients exhibited significant reductions (Post-intervention: B = 6.29, 95% CI: 3.03 to 9.54; Long-term follow-up: B = 8.11, 95% CI: 4.88 to 11.34). Similar trends were observed in DASS scores, with notable decreases both immediately after the intervention and sustained at follow-up (B = 6.56, 95% CI: 2.99 to 10.12 for both time points). In terms of social support, there was a surprising decrease post-intervention and at follow-up (Post-intervention: B = -3.71, 95% CI: -5.78 to -1.64; Long-term follow-up: B = -3.84, 95% CI: -6.08 to -1.59), suggesting a potential area for further investigation. PTSD scores showed a significant reduction following the intervention, with sustained improvements at follow-up (Post-intervention: B = 9.56, 95% CI: 7.70 to 11.50; Long-term follow-up: B = 8.61, 95% CI: 6.46 to 10.77). Lastly, client satisfaction post-intervention indicated a slight negative shift (B = -0.77, 95% CI: -1.18 to -0.36), warranting a closer examination of the factors contributing to this outcome. Each of these models was carefully adjusted for age, gender, education, and baseline scores on the respective outcomes, ensuring a robust analysis of the IA-PM+'s impact.

**Supplementary Table 1**

| Effectiveness of IA-PM+ on Disability Post-Intervention: Linear Mixed Effects Model Results  *Estimates of Fixed Effects^a^* | | | | | | | |
| --- | --- | --- | --- | --- | --- | --- | --- |
| Parameter | Estimate | Std. Error | df | t | Sig. | 95% Confidence Interval | |
|  |  |  |  |  |  | Lower Bound | Upper Bound |
| Intercept | 2.709 | 3.209 | 126.697 | .844 | .400 | -3.641 | 9.058 |
| Age | -.117 | .058 | 141.604 | -2.029 | .044 | -.231 | -.003 |
| Gender | 2.406 | 1.273 | 139.623 | 1.890 | .061 | -.111 | 4.922 |
| Education | .017 | .211 | 140.694 | .083 | .934 | -.399 | .434 |
| WHODAS at baseline | .727 | .073 | 138.727 | 9.907 | <.001 | .582 | .872 |
| Intervention Effect (B) | 6.286 | 1.331 | 6.027 | 4.723 | .003 | 3.033 | 9.540 |
|  |  |  |  |  |  | . | . |

Table 1 presents the results of the linear mixed effects model, showing that among IA-PM+ intervention recipients, disability scores (measured by WHODAS) significantly reduced after the intervention (Intervention Effect: B = -6.29, 95% CI: -9.54 to -3.03). This improvement was sustained at long-term follow-up (Intervention Effect: B = -8.11, 95% CI: -11.34 to -4.88). Intervention Effect (B) represents the estimated reduction in disability scores attributable to the IA-PM+ intervention, adjusted for baseline scores and participant characteristics, including age, gender, and education. Full results, including parameter estimates for other covariates, are provided in Supplementary Tables 13 and 14.

**Supplementary Table 2:** *Effectiveness of IA-PM+ on Disability at Long-Term Follow-Up: Linear Mixed Effects Model Results*

| *Estimates of Fixed Effects^a^* | | | | | | | |
| --- | --- | --- | --- | --- | --- | --- | --- |
| Parameter | Estimate | Std. Error | Df | t | Sig. | 95% Confidence Interval | |
|  |  |  |  |  |  | Lower Bound | Upper Bound |
| Intercept | -5.435 | 3.325 | 128.610 | -1.635 | .105 | -12.014 | 1.143 |
| Age | -.120 | .060 | 141.313 | -2.001 | .047 | -.238 | -.001 |
| Gender | 3.196 | 1.323 | 139.911 | 2.415 | .017 | .580 | 5.813 |
| Education | .004 | .219 | 140.954 | .018 | .986 | -.429 | .437 |
| WHODAS at baseline | .837 | .076 | 139.054 | 10.971 | <.001 | .686 | .987 |
| Intervention Effect (B) | 8.107 | 1.336 | 6.308 | 6.070 | <.001 | 4.877 | 11.337 |
|  |  |  | . | . | . | . | . |
| 1. Dependent Variable: WHODAS.   Supplementary Table 2 presents the results of the linear mixed effects model, demonstrating that among IA-PM+ intervention recipients, disability scores (measured by WHODAS) significantly reduced post-intervention (Intervention Effect: B = -6.29, 95% CI: -9.54 to -3.03). This reduction was sustained at long-term follow-up, with a further improvement in scores (Intervention Effect: B = -8.11, 95% CI: -11.34 to -4.88). The models were adjusted for baseline WHODAS scores as well as participant characteristics, including age, gender, and education, to ensure robust estimates. Comprehensive results and additional parameter estimates are detailed in Supplementary Tables 13 and 14. | | | | | | | |

**Supplementary Table 3: Effectiveness of IA-PM+ in Reducing DASS Scores Post-Intervention: Results from Linear Mixed Effects Models**

| *Estimates of Fixed Effects^a^* | | | | | | | |
| --- | --- | --- | --- | --- | --- | --- | --- |
| Parameter | Estimate | Std. Error | df | t | Sig. | 95% Confidence Interval | |
|  |  |  |  |  |  | Lower Bound | Upper Bound |
| Intercept | 2.614 | 3.470 | 126.629 | .753 | .453 | -4.252 | 9.480 |
| Age | -.059 | .065 | 139.931 | -.915 | .362 | -.187 | .069 |
| Gender | 2.759 | 1.455 | 139.780 | 1.896 | .060 | -.118 | 5.635 |
| Education | .153 | .243 | 141.180 | .628 | .531 | -.327 | .633 |
| Intervention Effect (B) | 6.555 | 1.391 | 5.052 | 4.711 | .005 | 2.989 | 10.121 |
| DASS | .550 | .062 | 141.463 | 8.844 | <.001 | .427 | .673 |
| a. Dependent Variable: DASS. | | | | | | | |

Supplementary Table 3 presents the results of the linear mixed effects model, showing that among IA-PM+ intervention recipients, scores on DASS assessments significantly reduced after the intervention (Intervention Effect: B = -6.56, 95% CI: -10.12 to -2.99) (see Table 15). This reduction in DASS scores was sustained at long-term follow-up (Intervention Effect: B = -6.56, 95% CI: -10.12 to -2.99) (see Table 16). The models were adjusted for age, gender, education, and baseline DASS scores to ensure robust estimation of the intervention effect.

**Supplementary Table 4:** Effectiveness of IA-PM+ in Sustaining Reductions in DASS Scores at Long-Term Follow-Up: Results from Linear Mixed Effects Models

| *Estimates of Fixed Effects^a^* | | | | | | | |
| --- | --- | --- | --- | --- | --- | --- | --- |
| **Parameter** | **Estimate** | **Std. Error** | **Df** | **t** | **Sig.** | **95% Confidence Interval** | |
|  |  |  |  |  |  | **Lower Bound** | **Upper Bound** |
| Intercept | 2.614 | 3.470 | 126.629 | .753 | .453 | -4.252 | 9.480 |
| Age | -.059 | .065 | 139.931 | -.915 | .362 | -.187 | .069 |
| Gender | 2.759 | 1.455 | 139.780 | 1.896 | .060 | -.118 | 5.635 |
| Education | .153 | .243 | 141.180 | .628 | .531 | -.327 | .633 |
| Intervention Effect (B) | 6.555 | 1.391 | 5.052 | 4.711 | .005 | 2.989 | 10.121 |
| DASS scores at baseline | .550 | .062 | 141.463 | 8.844 | <.001 | .427 | .673 |
| 1. Dependent Variable: DASS.   Supplementary Table 4 presents the results of linear mixed effects models, demonstrating that among IA-PM+ intervention recipients, scores on DASS assessments significantly reduced after the intervention (Intervention Effect: B = -6.56, 95% CI: -10.12 to -2.99) (see Table 15). This reduction in DASS scores was sustained at long-term follow-up (Intervention Effect: B = -6.56, 95% CI: -10.12 to -2.99) (see Table 16). The models were adjusted for age, gender, education, and baseline DASS scores to account for potential confounders and provide robust estimates of the intervention effect. | | | | | | | |

**Supplementary Table 5**

*Effectiveness of IA-PM+ in Enhancing Social Support Post-Intervention: Results from Linear Mixed Effects Models*

| *Estimates of Fixed Effects^a^* | | | | | | | |
| --- | --- | --- | --- | --- | --- | --- | --- |
| Parameter | Estimate | Std. Error | Df | t | Sig. | 95% Confidence Interval | |
|  |  |  |  |  |  | Lower Bound | Upper Bound |
| Intercept | 16.278 | 2.689 | 148 | 6.053 | <.001 | 10.964 | 21.592 |
| Age | .026 | .051 | 148 | .500 | .618 | -.076 | .127 |
| Gender | .127 | 1.163 | 148.000 | .109 | .913 | -2.171 | 2.426 |
| Education | -.336 | .195 | 148 | -1.726 | .086 | -.720 | .049 |
| Intervention Effect (B) | -3.710 | 1.049 | 148 | -3.538 | <.001 | -5.782 | -1.637 |
| MSPSS scores at baseline | .724 | .039 | 148 | 18.413 | <.001 | .646 | .802 |
| a. Dependent Variable: Perceived Social Support. | | | | | | | |

Supplementary Table 5 presents the results of linear mixed effects models, showing that among IA-PM+ intervention recipients, scores on social support assessments significantly decreased after the intervention (Intervention Effect: B = -3.71, 95% CI: -5.78 to -1.64) (see Table 17). This reduction in social support scores was sustained at long-term follow-up (Intervention Effect: B = -3.84, 95% CI: -6.08 to -1.59) (see Table 18). The models were adjusted for age, gender, education, and baseline social support scores to ensure robust estimation of the intervention effect.

**Supplementary Table 6: Effectiveness of IA-PM+ in Sustaining Improvements in Social Support at Long-Term Follow-Up: Results from Linear Mixed Effects Models**

| *Estimates of Fixed Effects^a^* | | | | | | | |
| --- | --- | --- | --- | --- | --- | --- | --- |
| Parameter | Estimate | Std. Error | df | t | Sig. | 95% Confidence Interval | |
|  |  |  |  |  |  | Lower Bound | Upper Bound |
| Intercept | 15.140 | 2.911 | 148 | 5.201 | <.001 | 9.388 | 20.893 |
| Age | -.009 | .056 | 148 | -.169 | .866 | -.119 | .101 |
| Gender | 2.131 | 1.259 | 148 | 1.692 | .093 | -.357 | 4.619 |
| Education | -.579 | .211 | 148 | -2.748 | .007 | -.995 | -.163 |
| Intervention Effect (B) | -3.836 | 1.135 | 148 | -3.379 | <.001 | -6.079 | -1.592 |
| MSPSS at baseline | .763 | .043 | 148 | 17.918 | <.001 | .679 | .847 |
| 1. Dependent Variable: Perceived Social Support.   Supplementary Table 6 presents the results of linear mixed effects models, showing that among IA-PM+ intervention recipients, scores on social support assessments significantly decreased after the intervention (Intervention Effect: B = -3.71, 95% CI: -5.78 to -1.64) (see Table 17). This reduction in social support scores was sustained at long-term follow-up (Intervention Effect: B = -3.84, 95% CI: -6.08 to -1.59) (see Table 18). The models were adjusted for age, gender, education, and baseline social support scores to account for potential confounders and ensure robust estimates of the intervention effect. | | | | | | | |

**Supplementary Table 7: Effectiveness of IA-PM+ in Reducing PTSD Symptoms Post-Intervention: Results from Linear Mixed Effects Models**

| *Estimates of Fixed Effects^a^* | | | | | | | |
| --- | --- | --- | --- | --- | --- | --- | --- |
| Parameter | Estimate | Std. Error | df | t | Sig. | 95% Confidence Interval | |
|  |  |  |  |  |  | Lower Bound | Upper Bound |
| Intercept | -2.531 | 2.082 | 148.000 | -1.215 | .226 | -6.646 | 1.585 |
| Age | .131 | .047 | 148.000 | 2.777 | .006 | .038 | .224 |
| Gender | -3.901 | 1.065 | 148.000 | -3.661 | <.001 | -6.006 | -1.795 |
| Education | .026 | .183 | 148 | .144 | .886 | -.335 | .388 |
| Intervention Effect (B) | 9.599 | .961 | 148 | 9.992 | <.001 | 7.701 | 11.498 |
| PTSD at baseline | .802 | .040 | 148 | 20.043 | <.001 | .723 | .881 |
| a. Dependent Variable: PTSD. | | | | | | | |

Supplementary Table 7 presents the results of linear mixed effects models, showing that among IA-PM+ intervention recipients, scores on PTSD assessments significantly decreased after the intervention (Intervention Effect: B = -9.56, 95% CI: -11.50 to -7.70) (see Table 19). This reduction in PTSD scores was sustained at long-term follow-up (Intervention Effect: B = -8.61, 95% CI: -10.77 to -6.46) (see Table 20). The models were adjusted for age, gender, education, and baseline PTSD scores to account for potential confounders and provide robust estimates of the intervention effect.

**Supplementary Table 8: Effectiveness of IA-PM+ in Sustaining Reductions in PTSD Symptoms at Follow-Up: Results from Linear Mixed Effects Models**

| *Estimates of Fixed Effects^a^* | | | | | | | |
| --- | --- | --- | --- | --- | --- | --- | --- |
| Parameter | Estimate | Std. Error | df | t | Sig. | 95% Confidence Interval | |
|  |  |  |  |  |  | Lower Bound | Upper Bound |
| Intercept | -.420 | 2.364 | 148 | -.178 | .859 | -5.091 | 4.251 |
| Age | .156 | .054 | 148 | 2.907 | .004 | .050 | .262 |
| Gender | -5.917 | 1.209 | 148 | -4.893 | <.001 | -8.307 | -3.527 |
| Education | -.192 | .208 | 148 | -.925 | .357 | -.603 | .218 |
| Intervention Effect (B) | 8.610 | 1.090 | 148 | 7.896 | <.001 | 6.455 | 10.765 |
| PTSD at baseline | .771 | .045 | 148 | 16.980 | <.001 | .681 | .860 |
| 1. Dependent Variable: PTSD.   Supplementary Table 8 presents the results of linear mixed effects models, showing that among IA-PM+ intervention recipients, scores on PTSD assessments significantly decreased after the intervention (Intervention Effect: B = -9.56, 95% CI: -11.50 to -7.70) (see Table 19). This reduction in PTSD scores was sustained at long-term follow-up (Intervention Effect: B = -8.61, 95% CI: -10.77 to -6.46) (see Table 20). The models were adjusted for age, gender, education, and baseline PTSD scores to account for potential confounders and ensure robust estimates of the intervention effect. | | | | | | | |

**Supplementary Table 9: Linear Mixed Effects Models Evaluating Client Satisfaction Post-Intervention with IA-PM+**

| *Estimates of Fixed Effects^a^* | | | | | | | |
| --- | --- | --- | --- | --- | --- | --- | --- |
| Parameter | Estimate | Std. Error | df | t | Sig. | 95% Confidence Interval | |
|  |  |  |  |  |  | Lower Bound | Upper Bound |
| Intercept | 18.196 | .398 | 148 | 45.709 | <.001 | 17.410 | 18.983 |
| Age | -.012 | .010 | 148.000 | -1.139 | .257 | -.031 | .008 |
| Gender | .376 | .228 | 148.000 | 1.646 | .102 | -.075 | .827 |
| Education | -.031 | .038 | 148 | -.813 | .418 | -.105 | .044 |
| Intervention Effect (B) | -.769 | .206 | 148 | -3.723 | <.001 | -1.177 | -.361 |
| 1. Dependent Variable: Client Satisfaction.   Supplementary Table 9 presents the results of linear mixed effects models, showing that among IA-PM+ intervention recipients, scores on client satisfaction assessments significantly decreased after the intervention (Intervention Effect: B = -0.77, 95% CI: -1.18 to -0.36). The models were adjusted for age, gender, education, and baseline client satisfaction scores to account for potential confounders and provide robust estimates of the intervention effect. | | | | | | | |

**Supplementary table 10: Descriptive statistics of outcomes during baseline, postintervention and follow-up**

| **Outcomes** | **Groups** | **N** | **Mean** | **Std. Deviation** |
| --- | --- | --- | --- | --- |
| **Baseline** | | | | |
| GHQ | Control-TAU | 74 | 20.9054 | 6.31625 |
|  | Intervention-PM | 74 | 21.8378 | 6.71744 |
| WHODAS | Control-TAU | 74 | 35.9865 | 8.21207 |
|  | Intervention-PM | 74 | 36.3649 | 7.72396 |
| DASS | Control-TAU | 74 | 35.2162 | 10.89586 |
|  | Intervention-PM | 74 | 37.7297 | 10.33189 |
| Depression | Control-TAU | 74 | 11.5405 | 4.10876 |
|  | Intervention-PM | 74 | 12.4459 | 4.05490 |
| Anxiety | Control-TAU | 74 | 11.7432 | 3.74554 |
|  | Intervention-PM | 74 | 12.2162 | 3.18932 |
| Stress | Control-TAU | 74 | 11.9324 | 3.48905 |
|  | Intervention-PM | 74 | 13.0676 | 3.96329 |
| Perceived Social Support | Control-TAU | 74 | 46.2432 | 13.48344 |
|  | Intervention-PM | 74 | 46.8378 | 13.55863 |
| Life Satisfaction | Control-TAU | 74 | 18.5541 | 4.78337 |
|  | Intervention-PM | 74 | 16.5000 | 4.82644 |
| PTSD | Control-TAU | 74 | 20.8108 | 11.61444 |
|  | Intervention-PM | 74 | 21.7432 | 13.20550 |
| **Post-intervention** | | | | |
| WHODAS | Control-TAU | 74 | 34.2568 | 7.30371 |
|  | Intervention-PM | 74 | 28.3378 | 10.34643 |
| DASS | Control-TAU | 74 | 30.8243 | 10.30807 |
|  | Intervention-PM | 74 | 25.4324 | 9.29440 |
| Depression | Control-TAU | 74 | 10.1081 | 3.85194 |
|  | Intervention-PM | 74 | 8.5000 | 3.11096 |
| Anxiety | Control-TAU | 74 | 10.0541 | 3.36335 |
|  | Intervention-PM | 74 | 7.9595 | 3.11179 |
| Stress | Control-TAU | 74 | 10.6622 | 3.49668 |
|  | Intervention-PM | 74 | 8.9730 | 3.74887 |
| Perceived Social Support | Control-TAU | 74 | 46.2703 | 10.94481 |
|  | Intervention-PM | 74 | 50.4865 | 12.14439 |
| Life Satisfaction | Control-TAU | 74 | 18.5541 | 4.78337 |
|  | Intervention-PM | 74 | 16.5000 | 4.82644 |
| PTSD | Control-TAU | 74 | 23.0270 | 13.39799 |
|  | Intervention-PM | 74 | 14.2703 | 9.79837 |
| Client_Satisfaction | Control-TAU | 74 | 17.4595 | 1.25173 |
|  | Intervention-PM | 74 | 18.2297 | 1.27728 |
| **Follow up** | | | | |
| WHODAS | Control-TAU | 74 | 32.9459 | 7.66757 |
|  | Intervention-PM | 74 | 25.1486 | 11.49352 |
| DASS | Control-TAU | 74 | 30.8243 | 10.30807 |
|  | Intervention-PM | 74 | 25.4324 | 9.29440 |
| Depression | Control-TAU | 74 | 10.2297 | 3.84119 |
|  | Intervention-PM | 74 | 7.0135 | 3.17306 |
| Anxiety | Control-TAU | 74 | 9.6892 | 3.03372 |
|  | Intervention-PM | 74 | 7.0405 | 2.48524 |
| Stress | Control-TAU | 74 | 10.2703 | 3.21511 |
|  | Intervention-PM | 74 | 8.0676 | 3.62764 |
| Perceived Social Support | Control-TAU | 74 | 47.7568 | 10.46561 |
|  | Intervention-PM | 74 | 52.0946 | 13.75337 |
| Life Satisfaction | Control-TAU | 74 | 18.6351 | 4.06311 |
|  | Intervention-PM | 74 | 19.7568 | 3.88468 |
| PTSD | Control-TAU | 74 | 20.7838 | 13.20157 |
|  | Intervention-PM | 74 | 13.2703 | 10.96982 |

**Supplementary tables 11: Pre-post differences in outcome scores in the intervention group using dependent sample t-test**

| Outcome | Timepoint | Mean | N | Std. Deviation | Std. Error Mean | Post-intervention vs baseline | | Follow up vs baseline | |
| --- | --- | --- | --- | --- | --- | --- | --- | --- | --- |
|  |  |  |  |  |  | t-value | p-value | t-value | p-value |
| WHODAS | Baseline | 36.3649 | 74 | 7.72396 | .89789 | 10.151 | <0.001 |  |  |
|  | Post-intervention | 28.3378 | 74 | 10.34643 | 1.20275 |  |  |  |  |
|  | Follow up | 25.1486 | 74 | 11.49352 | 1.33609 |  |  | 13.761 | <0.001 |
| DASS | Baseline | 37.7297 | 74 | 10.33189 |  | 11.380 | <0.001 |  |  |
|  | Post-intervention | 25.4324 | 74 | 9.29440 | 1.08045 |  |  |  |  |
|  | Follow up | 25.4324 | 74 | 9.29440 | 1.08045 |  |  | 11.380 | <0.001 |
| Depression | Baseline | 12.4459 | 74 | 4.05490 | .47137 | 9.323 | <0.001 |  |  |
|  | Post-intervention | 8.5000 | 74 | 3.11096 | .36164 |  |  |  |  |
|  | Follow up | 7.0135 | 74 | 3.17306 | .36886 |  |  | 14.332 | <0.001 |
| Anxiety | Baseline | 12.2162 | 74 | 3.18932 | .37075 | 1.20106 | <0.001 |  |  |
|  | Post-intervention | 7.9595 | 74 | 3.11179 | .36174 |  |  |  |  |
|  | Follow up | 7.0405 | 74 | 2.48524 | .28890 |  |  | 15.786 | <0.001 |
| Stress | Baseline | 13.0676 | 74 | 3.96329 | .46072 | 10.979 | <0.001 |  |  |
|  | Post-intervention | 8.9730 | 74 | 3.74887 | .43580 |  |  |  |  |
|  | Follow up | 8.0676 | 74 | 3.62764 | .42170 |  |  | 11.473 | <0.001 |
| Perceived Social Support | Baseline | 46.8378 | 74 | 13.55863 | 1.57616 | 9.064 | <0.001 |  |  |
|  | Post-intervention | 50.4865 | 74 | 12.14439 | 1.41176 |  |  |  |  |
|  | Follow up | 52.0946 | 74 | 13.75337 | 1.59880 |  |  | -6.082 | <0.001 |
| Life Satisfaction | Baseline | 16.5000^a^ | 74 | 4.82644 | .56106 | -5.552 | <0.001 |  |  |
|  | Post-intervention | 16.5000^a^ | 74 | 4.82644 | .56106 |  |  |  |  |
|  | Follow up | 19.7568 | 74 | 3.88468 | .45158 |  |  | -8.032 | <0.001 |
| PTSD | Baseline | 21.7432 | 74 | 13.20550 | 1.53511 | 10.605 | <0.001 |  |  |
|  | Post-intervention | 14.2703 | 74 | 9.79837 | 1.13904 |  |  |  |  |
|  | Follow up | 13.2703 | 74 | 10.96982 | 1.27522 |  |  | 10.065 | <0.001 |
| a. The correlation and t cannot be computed because the standard error of the difference is 0. | | | | | | | |  |  |

**Supplementary Table 12: Responses to the client satisfaction questionnaire**

| **Statement** | **Response** | **Frequency** | **Percentage** |
| --- | --- | --- | --- |
| 1. How would you rate the quality of service you received? | Poor | 0 | 0.0% |
|  | Good | 101 | 68.2% |
|  | Fair | 47 | 31.8% |
|  | Excellent | 0 | 0.0% |
| 2. Did you get the kind of service you wanted? | Yes, definitely | 22 | 14.9% |
|  | Yes, generally | 122 | 82.4% |
|  | No, not really | 4 | 2.7% |
|  | No, definitely | 0 | 0.0% |
| 3. To what extent has our service met your needs? | None of my needs have been met | 0 | 0.0% |
|  | Only a few of my needs have been met | 83 | 56.1% |
|  | Most of my needs have been met | 49 | 33.1% |
|  | Almost all of my needs have been met | 16 | 10.8% |
| If your friend were in need of similar help, would you recommend our service to him or her? | Yes, definitely | 32 | 21.6% |
|  | Yes, I think so | 112 | 75.7% |
|  | No, I don’t think so | 4 | 2.7% |
|  | No, definitely not | 0 | 0.0% |
| 5. How satisfied are you with the amount of help you received? | Very satisfied | 15 | 10.1% |
|  | Mostly satisfied | 129 | 87.2% |
|  | Indifferent or mildly dissatisfied | 4 | 2.7% |
|  | Quite dissatisfied | 0 | 0.0% |
| 6. Have the services you received helped you to deal more effectively with your problems? | No, they seemed to make things worse | 0 | 0.0% |
|  | No, they really didn’t help | 44 | 29.7% |
|  | Yes, they helped somewhat | 72 | 48.6% |
|  | Yes, they helped a great deal | 32 | 21.6% |
| 7. In an overall, general sense, how satisfied are you with the service you received? | Very satisfied | 0 | 0.0% |
|  | Mostly satisfied | 35 | 23.6% |
|  | Indifferent of mildly dissatisfied | 113 | 76.4% |
|  | Quite dissatisfied | 0 | 0.0% |
| 8. If you were to seek help again, would you come back to our service? | Yes, definitely | 47 | 31.8% |
|  | Yes, I think so | 101 | 68.2% |
|  | No, I don’t think so | 0 | 0.0% |
|  | No, definitely not | 0 | 0.0% |
